# Generational Association Studies of Dopaminergic Genes in Reward Deficiency Syndrome (RDS) Subjects: Selecting Appropriate Phenotypes for Reward Dependence Behaviors

**DOI:** 10.3390/ijerph8124425

**Published:** 2011-11-29

**Authors:** Kenneth Blum, Amanda L. C. Chen, Marlene Oscar-Berman, Thomas J. H. Chen, Joel Lubar, Nancy White, Judith Lubar, Abdalla Bowirrat, Eric Braverman, John Schoolfield, Roger L. Waite, Bernard W. Downs, Margaret Madigan, David E. Comings, Caroline Davis, Mallory M. Kerner, Jennifer Knopf, Tomas Palomo, John J. Giordano, Siobhan A. Morse, Frank Fornari, Debmalya Barh, John Femino, John A. Bailey

**Affiliations:** 1 Department of Psychiatry, School of Medicine and McKnight Brain Institute, University of Florida, W University Ave., Gainesville, FL 32601, USA; Email: baileyjo@ufl.edu; 2 Department of Engineering Management Advanced Technology, Chang Jung Christian University, No. 396, Sec. 1, Changrong Road, Tainan 71101, Taiwan; 3 Department of Anatomy & Neurobiology, Boston University School of Medicine, 72 East Concord Street, Boston, MA 02118, USA; Email: Oscar@bu.edu; 4 Department of Occupational Safety and Health, Chang Jung Christian University, No. 396, Sec. 1, Changrong Road, Tainan 71101, Taiwan; Email:tjhchen@yahoo.com.tw; 5 Emeritus, Department of Physiology, University of Tennessee, 719 Andy Holt Tower, Knoxville, TN 37996, USA; Email: lubar@utkux.utcc.utk.edu; 6 Unique Mindcare, Inc., 1900 Saint James Place, Houston, TX 77056, USA; Email: nancy@Uniquemindcare.com; 7 Department of Neurofeedback, Southeastern Biofeedback and Neurobehavioral Clinic, 101 Westwood Road, Knoxville, TN 37919, USA; Email: lubar@utkux.utcc.utk.edu; 8 Department of Neuroscience & Population Genetics, EMMS Nazareth Hospital, Nazareth, Israel; Email: bowirrat@netvision.net.il; 9 Department of Neurosurgery, Weill Cornell College of Medicine, 1300 York Ave., New York, NY 10065, USA; Email: pathfoundationny@gmail.com; 10 Department of Academic Informatics Services, University of Texas Health Science Center, 7703 Floyd Curl Drive, San Antonio, TX 78229, USA; Email: schoolfield@uthscsa.edu; 11 Department of Nutrigenomics, LifeGen, Inc., P.O. Box 366, 570 Lederach Stattion Way, Lederach, PA 19450, USA; Email: drw8@san.rr.com (R.L.W.); bernardwdowns@gmail.com (B.W.D.); MARGBETTS@hotmail.com (M.M.); 12 Department of Genomic Research, Carlsbad Science Foundation, Department of Medical Genetics, City of Hope National Medical Center, 1500 Duarte Road, Duarte, CA 91010, USA; Email: dcomings@earthlink.net; 13 Department of Kinesiology and Health Sciences, York University, 4700 Keele Street, Toronto, ON M3J 1P3, Canada; Email: cdavis@yorku.ca; 14 Department of Integrative Medicine, PATH Medical Research Foundation, 304 Park Ave. South, New York, NY 10010, USA; Email: Mallory.kerner@gmail.com (M.M.K.); info@pathfoundationny.org (J.K.); 15 Hospital Universitario 12 de Octubre, Servicio de Psiquiatria, Av. Cordoba SN, Madrid 28041, Spain; Email: tpalomo2004@yahoo.es; 16 Department of Holistic Medicine, G&G Holistic Addiction Treatment, Inc., 1590 Northeast 162nd Street, North Miami Beach, FL 33162, USA; Email: michg8@hotmail.com; 17 Department of Research, National Institute for Holistic Addiction Studies, 1590 Northeast 162nd Street, North Miami Beach, FL 33162, USA; Email: samorse10@aol.com; 18 Dominion Diagnostics, Inc., 211 Circuit Road, North Kingstown, RI 02852, USA; Email: ffornari@me.com; 19 Center for Genomics and Applied Gene Technology, Institute of Integrative Omics and Applied Biotechnology, Nonakuri, Purba Medinipur, West Bengal, India; Email: dr.barh@gmail.com; 20 Meadows Edge Recovery Center, 580 10 Rod Road, North Kingstown, RI 02852, USA; Email: jfemino@meadowsedge.com

**Keywords:** dopamine, gene polymorphisms, generational association studies, phenotype, “super normal” controls, Reward Deficiency Syndrome (RDS)

## Abstract

Abnormal behaviors involving dopaminergic gene polymorphisms often reflect an insufficiency of usual feelings of satisfaction, or Reward Deficiency Syndrome (RDS). RDS results from a dysfunction in the “brain reward cascade,” a complex interaction among neurotransmitters (primarily dopaminergic and opioidergic). Individuals with a family history of alcoholism or other addictions may be born with a deficiency in the ability to produce or use these neurotransmitters. Exposure to prolonged periods of stress and alcohol or other substances also can lead to a corruption of the brain reward cascade function. We evaluated the potential association of four variants of dopaminergic candidate genes in RDS (dopamine D1 receptor gene [DRD1]; dopamine D2 receptor gene [DRD2]; dopamine transporter gene [DAT1]; dopamine beta-hydroxylase gene [DBH]). *Methodology*: We genotyped an experimental group of 55 subjects derived from up to five generations of two independent multiple-affected families compared to rigorously screened control subjects (e.g., N = 30 super controls for DRD2 gene polymorphisms). Data related to RDS behaviors were collected on these subjects plus 13 deceased family members. *Results*: Among the genotyped family members, the DRD2 Taq1 and the DAT1 10/10 alleles were significantly (at least p < 0.015) more often found in the RDS families *vs*. controls. The TaqA1 allele occurred in 100% of Family A individuals (N = 32) and 47.8% of Family B subjects (11 of 23). No significant differences were found between the experimental and control positive rates for the other variants. *Conclusions*: Although our sample size was limited, and linkage analysis is necessary, the results support the putative role of dopaminergic polymorphisms in RDS behaviors. This study shows the importance of a nonspecific RDS phenotype and informs an understanding of how evaluating single subset behaviors of RDS may lead to spurious results. Utilization of a nonspecific “reward” phenotype may be a paradigm shift in future association and linkage studies involving dopaminergic polymorphisms and other neurotransmitter gene candidates.

## 1. Introduction

### 1.1. Background

Reward Deficiency Syndrome (RDS) was first defined by our lab in 1996 as a putative predictor of impulsive and addictive behaviors [1,2,3,4,5,6,7]. It refers to an absence of usual feelings of satisfaction and a failure of the system that normally confers gratification, resulting in behaviors such as overeating, heavy cigarette smoking, drug and alcohol abuse, gambling, and hyperactivity (see Table 1 in a later section). The syndrome has been linked to dysfunction of dopamine (DA) receptors, the genes for which show many mutant forms. In an attempt to resolve controversy regarding the causal contributions of mesolimbic DA systems to reward, we have evaluated the three main competing explanatory categories: “liking,” “learning,” and “wanting” [8], especially as they relate to RDS [9]. That is, DA may mediate: (a) the hedonic impact of reward (liking), (b) learned predictions about rewarding effects (learning), or (c) the pursuit of rewards by attributing incentive salience to reward-related stimuli (wanting). We evaluated these hypotheses, especially as they relate to the Reward Deficiency Syndrome (RDS), and we find that the incentive salience or “wanting” hypothesis of DA function is supported by a majority of the evidence [9]. 

Neuroimaging studies have shown that drugs of abuse, palatable foods, and anticipated behaviors such as sex and gaming affect brain regions involving reward circuitry, and may not be unidirectional. Gardner [10] has suggested that drugs of abuse that promote DA signals short circuit and sensitize dynamic mesolimbic mechanisms that evolved to attribute incentive salience to rewards. Accordingly, Gardner [10] further suggested that addictive drugs have in common that they are voluntarily self-administered, they enhance (directly or indirectly) dopaminergic synaptic function in the nucleus accumbens (NAc), and they stimulate the functioning of brain reward circuitry (producing the “high” that drug users seek). Blum and Gold [11] pointed out that reward circuitry is very complex, especially as these circuits relate to hedonic tone. Moreover, these circuits now are believed to be functionally more complex, also encoding attention, reward expectancy, disconfirmation of reward expectancy, and incentive motivation. Elevated stress levels, together with polymorphisms of dopaminergic genes and other neurotransmitter genetic variants, may have a cumulative effect on vulnerability to addiction. We and others believe that the RDS model of etiology holds very well for addictions [10].

The D2 receptor has been associated with pleasure, and the DRD2 has been referred to as a reward gene [9,10,11,12,13,14,15,16]. The DRD2 gene, and especially the Taq1 A1 allele, has been associated with neuropsychiatric disorders in general, including alcoholism, other addictions (e.g., carbohydrate) [17,18,19,20,21,22,23], and it also may be involved in co-morbid antisocial personality disorder symptoms [24], especially in children and adults with attention deficit hyperactivity disorder (ADHD) or Tourette Syndrome [5,25] and high novelty seeking [26,27,28,29].

DA is involved in a remarkable number of behavioral functions including cognition and motor effects [30], depending upon its localization in the brain. DA has been called the “anti-stress molecule” and/or the “pleasure molecule” [2,31,32,33] and is released into the synapse in the NAc where it stimulates a number of receptors (D1–D5), which results in increased feelings of well-being and stress reduction. The mesocorticolimbic dopaminergic pathway plays an especially important role in mediating the reinforcement of natural rewards like food and sex, as well as unnatural rewards like drugs of abuse. Natural rewards include satisfaction of physiological drives (e.g., hunger and reproduction) [34,35], and unnatural rewards are learned and involve satisfaction of acquired pleasures such as hedonic sensations [36,37] derived from alcohol and other drugs, as well as from gambling and other risk-taking behaviors [33,34,35,36,38,39]. 

In discussing RDS, we refer specifically to an insensitivity and inefficiency in the reward system [1,2,3,4,40,41]. There may be a common neurocircuitry [42] and neurobiology [43] for multiple addictions [44,45] and for a number of psychiatric disorders [43,44,45,46,47,48,49,50,51]. Due to specific genetic antecedents and environmental influences [52], a deficiency of the D2 receptors may predispose individuals to a high risk for multiple addictive, impulsive, and compulsive behaviors [4,31,53,54,55,56,57]. It is well known that alcohol and other drugs of abuse [58], as well as most positive reinforcers (e.g., sex [59], food [60], gambling [61,62,63], aggressive thrills [6]) cause activation and neuronal release of brain DA [32,62,63], which in turn can decrease negative feelings and satisfy abnormal cravings for alcohol, cocaine, heroin, and nicotine, and which are linked to low DA function [53,55,56,64,65,66,67,68,69,70,71,72,73,74,75,76,77,78]. 

Since there is a commonality in the mechanism by which drugs of abuse, smoking, food, sex, gaming—and their associated cues, e.g., seeing drug paraphernalia—stimulate the release of mesolimbic DA at the NAc, it is difficult to determine which, if any, of these RDS behaviors will specifically manifest in a family member. However, utilizing a Bayesian mathematical model, we have found that at least for carriers of the DRD2 A1 allele, the estimated predictive value is 74% [3]. Simply put, having this high predictive value suggests that an individual carrier could transfer one addiction for another [3]. In terms of gene expression at the mRNA level, there is specificity for many psychoactive drugs [79]. 

### 1.2. Brief Description of Risk Alleles in a Number of Dopaminergic Reward Genes

*Dopamine D2 receptor gene (DRD2)*. The DA D2 receptor gene (DRD2) first associated with severe alcoholism is the most widely studied gene in psychiatric genetics [19]. The Taq1 A is a single nucleotide polymorphism (SNP rs: 1800497) originally thought to be located in the 3'-untranslated region of the DRD2 but has since been shown to be located within exon 8 of an adjacent gene, the ankyrin repeat and kinase domain containing 1 (ANKK1). Importantly, while there may be distinct differences in function, the mis-location of the Taq1 A allele may be attributable to the ANKKI and the DRD2 being on the same haplotype or the ANKKI being involved in reward processing through a signal transduction pathway [80]. The ANKKI and the DRD2 gene polymorphisms may have distinct, different actions with regard to brain function [81]. Presence of the A1^+^ genotype (A1/A1, A1/A2) compared to the A^–^ genotype (A2/A2) is associated with reduced receptor density [82,83,84]. This reduction causes hypodopaminergic functioning in the DA reward pathway. Other DRD2 polymorphisms such as the C (57T, A SNP (rs: 6277) at exon 7 also associates with a number of RDS behaviors including drug use [85]. Compared to the T^–^ genotype (C/C), the T^+^ genotype (T/T, T/C) is associated with reduced translation of DRD2 mRNA and diminished DRD2 mRNA, leading to reduced DRD2 density and a predisposition to alcohol dependence [86]. The Taq1 A allele is a predictive risk allele in families [87]. 

*Dopamine transporter gene (DAT1).* The DA transporter protein regulates DA-mediated neurotransmission by rapidly accumulating DA that has been released into the synapse [88]. The DA transporter gene (SLC6A3 or DAT1) is localized to chromosome 5p15.3. Moreover, there is a VNTR polymorphism within the 3' non-coding region of DAT1 [89]. There are two important alleles that may independently increase risk for RDS behaviors. The 9 repeat (9R) VNTR has been shown to influence gene expression and to augment transcription of the DA transporter protein, resulting in an enhanced clearance of synaptic DA, yielding reduced levels of DA to activate postsynaptic neurons. Presence of the 9R VNTR has also been linked to Substance Use Disorder [87]. Moreover, in terms of RDS behaviors, tandem repeats of the DA transporter gene (DAT) [90] have been associated with high risk for ADHD in children and in adults alike [91,92]. The 10-repeat allele is significant for hyperactivity-impulsivity (HI) symptoms [93]. 

*Dopamine D1 receptor gene (DRD1).* Abnormalities in the dopaminergic reward pathways have frequently been implicated in substance abuse and addictive behaviors. Recent studies by Self [94] have suggested an important interaction between the DA D1 and D2 receptors in cocaine abuse. To test the hypothesis that the DRD1 gene might play a role in addictive behaviors Comings *et al.* [95], examined the alleles of the Dde I polymorphism in three independent groups of subjects with varying types of compulsive, addictive behaviors Tourette syndrome probands, smokers, and pathological gamblers. Specifically, in all three groups there was a significant representation of the frequency of homozygosity for the DRD1 Dde I 1 or 2 alleles in subjects with addictive behaviors. The DRD1 11 or 22 genotype was present in 41.3% of 63 controls and 57.3% of 227 TS probands (p = 0.024). When 23 quantitative traits were examined statistically, those carrying the 11 genotype consistently showed the highest scores. Based on these results, they examined the prevalence of the 11 genotype in controls and in Tourette syndrome probands. There was a highly significant progressive, linear increase in scores for gambling, alcohol use, and compulsive shopping. Problems with three additional behaviors, drug use, compulsive eating, and smoking also were significant. All six variables were related to addictive behaviors. In a totally separate group of controls and individuals attending a smoking cessation clinic, 39.3% of the controls *versus* 66.1% of the smokers carried the 11 or 22 genotype (p = 0.0002). In a third independent group of pathological gamblers, 55.8% carried the 11 or 22 genotype (p = 0.009 *versus* the combined controls). In the Tourette syndrome group and in smokers there was a significant additive effect of the DRD1 and DRD2 genes. The results for both the DRD1 and DRD2 genes, which have opposing effects on cyclic AMP, were consistent with negative and positive heterosis, respectively. These results support a role for genetic variants of the DRD1 gene in some addictive behaviors, and an interaction of genetic variants at the DRD1 and DRD2 genes. 

Volkow’s group [96], using *in vivo* optical microprobe imaging, tested the role of DA D1 receptors relative to DA D2 receptors during acute cocaine administration. Their results suggested that since activation of striatal D1R-expressing neurons (direct-pathway) enhanced cocaine reward, whereas activation of D2R-expressing neurons suppressed it (indirect-pathway), cocaine’s rewarding effects entailed both its fast stimulation of D1R (resulting in abrupt activation of direct-pathway neurons) and a slower stimulation of D2R (resulting in longer-lasting deactivation of indirect-pathway neurons). Lobo *et al*. also provided direct *in vivo* evidence of D2R and D1R optogenetic interactions in the striatal responses to acute cocaine administration [97].

*Dopamine beta-hydroxylase gene (DBH)*. DA β-hydroxylase (DBH) is a membrane-bound enzyme that converts DA to norepinephrine, thereby making norepinephrine and epinephrine the only transmitters synthesized inside vesicles (http://en.wikipedia.org/wiki/Dopamine_beta_hydroxylase). It is expressed in noradrenergic nerve terminals of the central and peripheral nervous systems, as well as in chromaffin cells of the adrenal medulla. Even prior to the emergence of the neuropsychogenetic field and candidate gene analysis was begun, Egeland identified a possible link to manic-depressive disorder and the tyrosine hydroxylase enzyme [98]. Subsequently, Comings and his colleagues [99] associated the DBH gene polymorphism with ADHD. Polymorphisms of three different dopaminergic genes, DA D2 receptor (DRD2), DBH, and DA transporter (DAT1), were examined in Tourette syndrome probands, their relatives, and controls. Each gene individually showed a significant correlation with various behavioral variables in these subjects. The additive and subtractive effects of the three genes were examined by genotyping all three genes in the same set of subjects. For nine of 20 Tourette syndrome associated comorbid behaviors, there was a significant linear association between the degree of loading for markers of three genes and the mean behavior scores. The behavior variables showing the significant associations were, in order: ADHD, stuttering oppositional defiant, tics, conduct, obsessive-compulsive, mania, alcohol abuse, and general anxiety. These are behaviors that constitute the most overt clinical aspects of Tourette syndrome. For 16 of the 20 behavior scores, there was a linear progressive decrease in the mean score, with progressively lesser loading for the three gene markers. 

In a recent PUBMED search, we coupled the terms DBH and ADHD, and we found 39 citations; the findings were both positive and negative. An interesting noteworthy example involves the work of Hess *et al.* [100]. Their findings did not implicate the DBH C-1021T polymorphism in the pathophysiology of depressive disorders or personality disorders, yet homozygosity at this locus appeared to increase the risk towards personality traits related to impulsiveness, aggression, and related disease states. In 2008, others [101] reported for polymorphisms G444A and C1603T in DBH, which were detected by univariant analysis, haplotype resulted in showing that the risk of ADHD was significantly increased in the presence of allele DBH +444A, as well as in the presence of allele DBH +1603T (O.R. = 15). Specifically, Barkley *et al.* [102] found that the DBH TaqI A2 allele, when homozygous, was associated with increased hyperactivity in childhood, pervasive behavior problems at adolescence, and earning less money on a card-playing task in adulthood. At adolescence, poorer test scores were also found only in the hyperactive group, which was homozygous for this allele. Similar associations with ADHD related behaviors have been reported by others [62,76,103], as well as in a meta-analysis showing the association of DBH and ADHD etiology [100]. Moreover, McKinney *et al*. [104] found that polymorphisms of DBH and MOA predicted whether a person was a heavy smoker and how many cigarettes they consumed. The findings of McKinney *et al*. [104] support the view that these enzymes help to determine a smoker’s requirement for nicotine and may explain why some people are predisposed to tobacco addiction and why some find it very difficult to stop smoking. 

We report here the results of the first intra-generational family association study, concerning a sampling of dopaminergic polymorphisms, utilizing a generalized RDS set of behaviors as the “phenotype” (see Table 1 defining thirds phenotype) rather than any single select phenotype, as well as a group of “super normal” control subjects. Super controls have been extensively screened for many associated RDS behaviors as defined herein (see below). Our results provide sufficient evidence to support a new approach to the study of Reward Deficient aberrant behaviors.

**Table 1:** Examples of behaviors and disorders [1,2,3,4,11,12,27,32,33,105,106,107,108,109,110,111,112,113,114,116,117,118,119,120,121,122,123,124,125,126,127,128,129,130,131,132,133,134,135,136,137,138,139,140,185,186,196,210,213] associated with Reward Deficiency Syndrome.

ADDICTIVE BEHAVIORS: Alcoholism; Drug Abuse; Smoking; Compulsive Eating and Obesity
IMPULSIVE BEHAVIORS: Attention Deficit Disorder; Attention Deficit Hyperactivity Disorder; Autistic Disorders; Tourette Syndrome
COMPULSIVE DISORDERS: Hypersexuality and Aberrant Sexual Behaviors; Pathological Gambling and Internet Gaming
PERSONALITY DISORDERS: Antisocial Personality Disorder; Conduct Disorder; Pathological Aggression; Generalized Anxiety Disorder

## 2. Methodology

**Figure 1:**
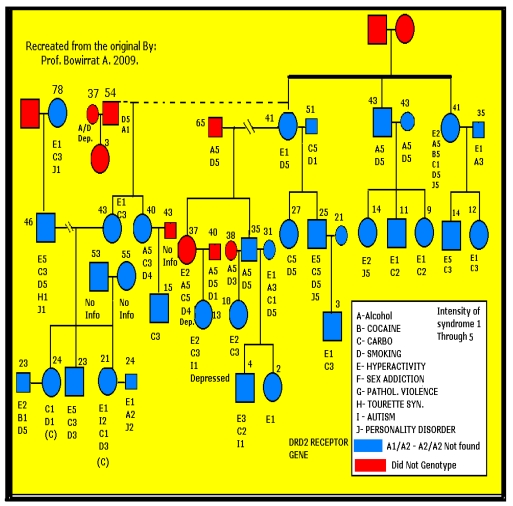
Genotype results of the Dopamine D2 receptor gene (DRD2) polymorphism of family A (n = 32) identified with multiple Reward Deficiency Syndrome (RDS) behaviors.

Although other neurotransmitter systems are involved in these complex behaviors representative of polygenic inheritance, we decided to evaluate the potential association of certain polymorphisms of the DA: D1 receptor (DRD1), DA D2 receptor (DRD2) [rs1800297 as a RFLP in “DRD2”] which it is near in the 3'-untranslated region contains ANKK1 on the opposite strand of DNA and our method herein targets the same site], DA transporter (DAT1), and DBH genes. To that end, we genotyped 55 subjects, from two independent multiple-affected families with documented RDS behaviors. There were four generations (Family A—initial proband was identified with ADHD) and five generations (Family B—initial probands was identified with substance use disorder). We had postmortem data related to RDS behaviors on all the deceased family members. A total of 13 members died, and their respective DNA specimens were not available. Figure 1 illustrates the genotyping and self-reported and family-identified behaviors for each family member.

### 2.1. Subject Selection

All individuals were evaluated through structured interviews using DMS-IV criteria and a number of neuropsychological and electrophysiological tests (e.g., Meyer-Briggs, Millon, TOVA, qEEG, *etc*.). The “super” control group consisted of 30 individuals selected from a total of 189 people attending PATH Medical Clinic, an integrative care center and research foundation in New York City, for both neurological and non-neurological problems (for more details, see [105]). These individuals were carefully screened, including their family history to exclude a number of RDS-related behaviors. The excluded behaviors included but where not limited to: alcoholism, substance use disorder, smoking behavior, carbohydrate binging, obesity, ADHD, posttraumatic stress disorder, conduct disorder, antisocial behavior, pathological gambling, aggressive offenses, pathological aggression, deviant sexual behavior, schizoid/avoidant behavioral cluster, and other Axis 1 and Axis 11 mental disorders. These subjects were genotyped for only the DRD2 gene polymorphisms (A1/A1, A1/A2. and A2/A2). In addition, we also genotyped 91 lesser screened controls (excluding only ADHD, pathological aggression, alcohol, tobacco, and other drug abuse and dependence) for the DAT1 9 and 10 alleles. Among these lesser-screened controls, 61 had DRD1 genotyping for the A1/A1, A2/A2, and A1/A2 alleles, and 51 had DBH genotyping for the B1/B1, B1/B2, and B2/B2 alleles. The study protocol was approved by the PATH Foundation IRB and ethics committee, and participants signed approved informed consent forms. 

**Figure 2:**
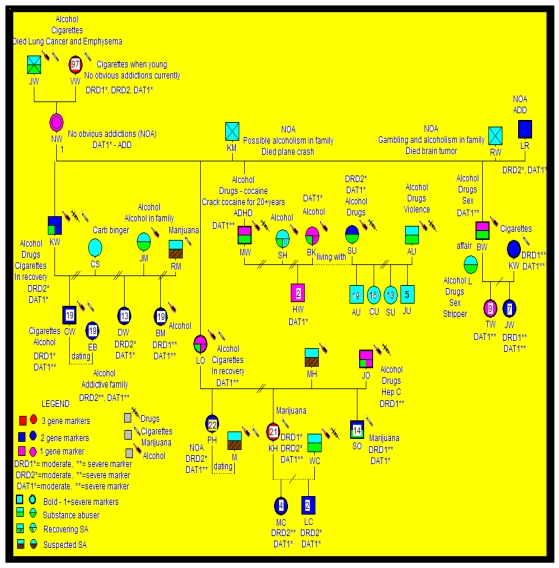
Association of dopaminergic gene polymorphisms with RDS behaviors in Family B (n = 23).

The experimental group consisted of 55 individuals from two independent families. Family A was provided for analysis by the Southeastern Biofeedback and Neurobehavioral Clinic, Knoxville, TN, and Family B was provided for analysis by the Enhancement Institute, Houston, TX (Unique Mind Care. Inc.) RDS diagnosis was available on 42 members from four generations of Family A (Figure 1) and 26 members from five generations of Family B (Table 2 and Figure 2). These included 10 deceased Family A members and three deceased Family B members. A total of 32 Family A and 23 Family B individuals were genotyped. In Family A, there were a total of 14 males and 18 females genotyped at an average age of 31.6 ± 18.9 years, and for Family B there were nine males and 14 females genotyped with average age of 31.5 ± 24.2 years. The members of Family A had been diagnosed with ADHD, and the members of Family B had been diagnosed with substance use disorder. The RDS behaviors observed included alcoholism, cocaine dependence, marijuana abuse, intravenous drug dependence, carbohydrate binging, obesity, smoking, hyperactivity, sex addiction, pathological aggression, Tourette syndrome, autism, criminal activity, gambling, novelty seeking, and personality disorders (see Figure 1 and Figure 2).

### 2.2. Genotyping

Buccal epithelial cells were collected by cotton swabs for DNA isolation. In some subjects, a blood sample was obtained. In this study we isolated the DNA and analyzed a number of genes. The genes include DRD1, DRD2, DAT1 and DBH. All subjects were genotyped based on a neutral identification number and read without knowledge of the individual being typed. Total genomic DNA was extracted from each coded blood/buccal sample, and aliquots were used for polymerase chain reaction (PCR) analysis. Genotyping was performed by a PCR technique. PCR was performed in 30-μL reaction mixtures containing 1.5 mM MgCl_2_, 2 mM 2'-deoxynucleotide 5'-triphosphates (dNTPs), 0.5 μMprimers, 1 μg of template DNA 1, 5 U of Taq polymerase (Boehringer Mannheim Corp., Indianapolis, IN, USA), and PCR buffer (20 mM Tris-HCL (pH 8.4) and 50 mM KCL. After an initial denaturation at 94 °C for 4 minutes, the DNA was amplified with 35 cycles of 30 seconds at 94 °C, 30 seconds at 58 °C, and 30 seconds at 72 °C, followed by a final extension step of 5 minutes at 72 °C.

*Dopamine D2 receptor gene.* The oligo-nucleotide primers 5'-CCGTCGACCCTTCCTGAGT-GTCATCA-3' and 5'-CCGTCGACGGCTGGCCAAGTTGTCTA-3' were used to amplify a 310-base pair fragment spanning the polymorphic TaqA1site of the DRD2 gene. The D2A1 and D2A2 genotyping was performed by a PCR technique. The PCR product was digested with 5 U of Taq 1 for 22 hours at 65 °C for the Taq1A polymorphism. Digestion products were then resolved on a 3% agarose gel (5 V/cm) containing 0.65 μg/mL ethidium bromide. There were three DRD2 Taq1A genotypes: the predominant homozygote A2/A2, which exhibits three restriction fragments of 180 and 130 bp; the heterozygote A1/A2, which exhibits three restriction fragments of 310, 180, and 130 bp; and the rare homozygote A1/A1, which produces only the uncleaved 310-bp fragment [20].

*Dopamine transporter gene.* DAT1 was genotyped by the technique of Comings *et al*. [5]. 

*Dopamine D1 receptor gene.* To examine the DRD1 gene we utilized the Dde I polymorphism consisting of an A to G change in the 5’ UTR, tested by the PCR procedure described by Thompson *et al*. [106]. 

*Dopamine beta-hydroxylase gene.* For DBH, the Taq I B polymorphism was genotyped by the technique of d’Amato *et al.* [107]. 

### 2.3. Statistical Analyses

Demographic, clinical, laboratory, interview, and questionnaire data were coded and entered into a computer database. DRD1, DRD2, DAT1 and DBH allelic prevalence, obtained by personnel blinded to the aforementioned information, also was coded. All comparisons were based on 2 × 2 contingency tables. Pearson’s chi-square statistic was used to identify group differences, with p < 0.05 considered statistically significant. In cases where at least one cell of the 2 × 2 contingency table had an expected frequency less than 5, Yates’ correction for continuity [108] was applied. To indicate the increase in odds of being gene positive for the experimental group relative to controls, odds ratios with 95% confidence intervals also were calculated. Group sample size of 55 for RDS and 30 for controls achieve 93% power to detect an odds ratio in the group porportions of 5.00 or more at the 0.05 level. All statistical analyses were performed using SPSS statistical software (SPSS, Inc., Chicago, IL, USA). 

## 3. Results

### 3.1. Genotyping

*Dopamine D2 receptor gene*. A large percentage (78.2) of the experimental subjects (43 of 55) carried the DRD2 Taq A1 allele. When compared with “super controls,” (1/30 or 3.3% of the controls carried the DRD2A1 allele), the experimental rate was significantly greater (χ^2^ = 43.6, p < 0.001) with an odds ratio of 103.9 (12.8, 843.2). Similarly, with regard to the DRD2 gene, when the experimental group (n = 55) was compared with unscreened literature controls derived from 15 international studies (LCN) [LCN = 439/3,329 (31.32%)], the experimental rate was significantly greater (χ^2^ = 187.1, p < 0.001) with an odds ratio of 23.6 (12.3, 45.1) (Table 2). Since the gene positive rate for Family B was lower than that for the combined families, separate experimental *vs*. control comparisons were made using Family B alone. When compared to “super controls”, Family B had a significantly greater positive rate (χ^2^ = 14.7, p < 0.001) with an odds ratio of 26.6 (3.1, 229.3), and a similar result was found when compared to unscreened literature controls (χ^2^ = 20.7, p < 0.001) with an odds ratio of 6.0 (2.6, 13.8). Based on over 3,000 unscreened subjects, the A1 allele of the DRD2 gene is present in approximately one-third of the American population, whereas the A2 allele is present in two-thirds of the American population [9]. 

*Dopamine transporter gene*. We found the less rigorous (no ADHD, alcoholism, substance abuse) 91 screened controls to carry the DAT1 10/10 in 34 subjects (37.4%); the 9/10 in 40 subjects (44.0%) and the 9/9 in 12 subjects (13.2%). With regard to the DAT1 gene, the 10/10 allele was present in 32 of 55 (58.2%) experimental group individuals in the two families. The experimental positive rate for the 10/10 allele was significantly greater (χ^2^ = 6.0, p < 0.015) with an odds ratio of 2.3 (1.2, 4.6). Moreover, 19 of the 32 probands (59.4%) from Family A (four generations) that were genotyped for the DAT1 gene carried the 10/10 allele, so that Family A had a significantly greater positive rate (χ^2^ = 4.7, p < 0.035) compared to controls with an odds ratio of 2.5 (1.1, 5.6). In Family B, 57.5% (13 of 23) carried the 10/10 genotype (Table 2 and Figure 1), but owing to the smaller sample size, the Family B positive rate was not significantly different (χ^2^ = 2.8, p = 0.095) from controls. Based on genotype data for the DAT1 gene on 3,080 subjects, the 480 bp 10/10 allele occurs in approximately 55% of the unscreened American population; the 9/10 occurs in approximately 38% of unscreened Americans; and the rare 9/9 allele occurs in 7% of unscreened Americans [5]. 

*Dopamine D1 receptor gene*. Because Comings *et al*. had shown the DA D1 receptor gene to be associated with substance use disorder [95], we analyzed this gene in Family B. When we compared subjects in Family B genotyped for polymorphisms of this gene with 61 controls, we found that in the Family B probands, 65.2 % carried the A1/A2 (15/23), 26.1% the A2/A2 (6/23), and 8.7% the A1/A1 (2/23) genotype (combined homozygosity A1/A1 and A2/A2 = 34.8%). When we compared the experimental homozygosity genotyping result against the 61 controls, for which three (4.9%) were positive for A1/A1, 20 (32.8%) were positive for A2/A2, and the combined homozygosity (A1/A1 and A2/A2) rate was 37.7%, no significant differences were observed (p > 0.50). In the American population, genotyping data revealed that 6% carry the A1/A1 allele (moderately dysfunctional); 35% carry the A2/A2 allele (severe dysfunction); and 60% carry the A1/A2 allele (normal) [5]. 

*Dopamine beta-hydroxylase gene*. Comparing the percent prevalence of the DA Beta-hydroxylase gene (DBHB1) in 51 controls [52.9 % (27/51)] with the 32 members genotyped only in Family A [65% (21/32)], DBHB1 was not significantly different (χ^2^ = 1.3, p > 0.25) (see Table 2). Since we found no significance with this gene in Family A, we decided not to test it in Family B. In the unscreened American population, genotyping data revealed that 21% carry the B1/B1 genotype, 69% carry the B1/B2 genotype and 58% carry the B2/B2 genotype [23,99]. However, the percentage decreases when one screens for no alcohol, drug or tobacco abuse/dependence (B1/B1 = 6%; B1/B2 = 21%; B2/B2 = 24%). 

### 3.2. Findings and Their Implications

Table 2 summarizes the genotype results for dopaminergic genes in Families A and B. The results for each gene polymorphism are presented. 

**Table 2:** Genotype results for dopaminergic genes in Family A and Family B.

Gene and Polymorphism	Percent Prevalence in Non-RDS Group	Percent Prevalence in Super Control Group	Percent Prevalence in RDS Group	Significance level p Value
**DRD2-A1 Allele**	31.32 (n = 3,143) *	3.3 (n = 30) **	78.2 (n = 55)	* p < 0.001
			Family A and B	** p < 0.001
**DAT1-10/10 Allele**	37.4 (n = 91) *	Not Applicable	58.18 (n = 55)	*p < 0.015
			Family A and B	
**DBH-B1 Allele**	52.9 (n = 51)	Not Applicable	65.0 (n = 32)	Not significant
			Family A	
**DRD1-A1/A1**	65.2 (n = 61)	Not Applicable	31.0 (n = 23)	Not significant
			Family B	

*Evaluating severity: Genetic Addiction Risk Score (GARS)*. Interestingly, in Family A, 100% of the subjects had at least one dysfunctional dopaminergic polymorphism. However, in considering the role of dopaminergic gene polymorphisms in RDS behavior, unlike Family A, where 100% of the subjects carried the DRD2 A1 allele, in Family B only 47.8% carried the TaqA1. Therefore, for Family B only, we evaluated severity by utilizing a Genetic Addiction Risk Score (GARS), a multivariate genetic index score methodology (developed by LifeGen, Inc. and Dominion Diagnostics, Inc.; see [109]) to strengthen the predictive value of laboratory testing for genetic predispositions related to disease diagnosis, stratification, prognosis, metabolism, and nutritional response. In this regard, the breakdown of the polymorphic markers was as follows: two subjects carried three polymorphic markers; 13 subjects had two polymorphic markers and eight subjects carried one polymorphic marker. 

## 4. Discussion

### 4.1. RDS, a Putative Endophenotype: Multiple Behaviors versus a Single Subset

In doing association studies for which an investigator requires a representative control sample for a single RDS psychiatric diagnosis [18,22,24,110,111,112] or for potential subsets of RDS (see Table 3), the obvious limitation relates to controls poorly screened for multiple RDS behaviors and other related psychiatric disorders.

Missing behaviors that are part of the RDS subset may be the reason for spurious results [9,87,113,114,115,116,117] when genotyping for single subsets of RDS behaviors. For example an individual may not drink or use drugs but may have other RDS behaviors like overeating or intensive video gaming. In support of this notion, we found a very strong association of the DA D2 receptor A1 allele (100%) in Family A. In addition, every individual in Family B also has at least one dopaminergic high risk allele (100%) [48% carried the DRD2 A1 allele]. Moreover, in Family B only three adult individuals had no addictive behaviors. When we compared our results in which 55 RDS subjects carried the DRD2 A1 allele at (78.2%) with the results of research by Noble [14] in which 597 severe alcoholic-dependent individuals (49.3%) carried the A1 allele, there was a significant difference between these two groups (χ^2^ = 16.9, p < 0.001). This demonstrated that the A1allele prevalence increases with multiple RDS behaviors. Here we propose that multifaceted non-specific RDS behaviors should be considered as the true “reward” phenotype (endophenotype) instead of a single subset RDS behavior such as alcoholism. Indeed, this may be a paradigm shift in future association and linkage studies using a different set of statistical methodologies.

**Table 3:** Correlation of RDS and related neurological and psychiatric disorders with DRD2 SNP (a sampling).

Reward Deficiency Syndrome or Related Disorder	Studies Demonstrating an Association with DRD2 Gene Polymorphism(s)
Pathological Gambling	[4,5,33,61,62,63,64,140]
Attention Deficit Hyperactivity Disorder	[2,4,24,99,154,196,207,208,209,210]
Post-Traumatic Stress Disorder	[4,176,177,196,212,213]
Eating; Obesity and Related Sequela	[46,133,134,170,178,179,181,182,197,198,200]
Energy Production	[134,180]
Hypertension	[181,182]
Schizophrenia	[12,19,49,112,183]
Early-Onset Sexual Intercourse; Hyper sexuality	[136,203]
Anti-Social Personality	[18,53]
Pathological Aggression	[6,105,171,186]
Schizoid-Avoidant Behavior	[143]
Novelty or Sensation Seeking	[26,27,36,83,109,113,144,161]
Substance Abuse	[12,13,20,22,29,46,65,71,90,103,110,114,117,172,173,174,175,193,199,201,202,205]
Heroin Addiction	[13,48,66,111,160]
Nicotine Dependence and Smoking Behavior	[13,17,22,81,91,104,184,185]
Personality Disorders and Crime	[100,186]
Parkinson’s Disease	[187,188]
Migraine	[189]
Tourette Syndrome	[5,99,190]
Huntington’s Disease	[191]
Cell Metabolism	[47]
Major Psychoses & Affective Disorder	[192,195,204]
Extraversion and Creativity	[21,23,145]

### 4.2. “Super” Controls as a Phenotype: Exclusion of Multiple RDS Behaviors

For population-based studies in which the investigator requires a representative control sample, removing confounding cases from the control group may improve chances of finding significant differences between experimental and control groups. This approach, however, may risk a lack of representation in the control group. Even the use of stratified samples (weighting samples) may not be good enough [113,114,115,116].

In the case of finding a “pure” phenotype, especially in the psychiatric arena, we really do not know if nature carved out the psychiatric disorders in the same fashion as is seen in the DSM. This is true because behavior is very complex, whereby specific genes for behavioral tendencies (anxiety, impulsivity, compulsivity, harm avoidance, aggressiveness, addiction *etc*.) accounts for only a small risk contribution to the overall phenotype. Therefore, we must shift our emphasis to the “systems biological” approach, which takes into account the inter relationship of dysfunctional behaviors, the polygenic nature of psychiatric disorders, and the environment. 

We should consider the established concept of RDS [1,2,3,4] to help define this complex array of behaviors associated with molecular dysfunctions. Victims of RDS are at increased vulnerability to addictive activities because they may carry polymorphic genes in dopaminergic pathways [117,118,119,120,121,122] that result in hypo-dopominergic function [60,61,62] caused by a reduced number of DA D2 receptors [55,117,118], reduced synthesis of DA (DA beta-hydroxylase) [5], reduced net release of pre-synaptic DA, possibly due to altered synthesis of DA (L-amino-acid decarboxylase) [123], increased synaptic clearance due to a high number of DA transporter sites [92,93,94,118,120,121,122] (DA transporter), and low D2 receptor densities [55,56,85,123,124,125,126,127] (DA D2 receptor). The need for a unified set of related symptoms in the affected phenotype is important not only for population-based association studies, but also for linkage analysis. The RDS concept involves, shared genes and behavioral tendencies as summarized in Table 3*.* While poly-genes are involved [128,129,130,131,132,133,134,135,136,137,138,139,140,141,142,143,144,145], the common theme in all of these substances and behaviors is that they induce pre-synaptic DA release at the NAc [53,73,74,75,76,77]. Spectrum disorders such as ADHD, Tourette Syndrome, and autism are included due to DA dysregulation [1,5,24,25]. In fact love styles also associate with both serotonergic and dopaminergic gene polymorphisms [146]. 

A screened control group is essential for uncovering population-based associations where the disease in question may be very common. We know that approximately one-third of the population meet lifetime criteria for common psychiatric disorders according to the results of the Epidemiological Catchment Area survey. Since RDS is a “polygenic disorder” involving multiple genes and many polymorphisms [1,2,3,4] and requires a threshold number of polygenes, unaffected individuals in the population also carry some of these genes. AS stated earlier, the DA D2 receptor gene (A1 allele) is present in about one-third of unscreened Americans (29.4% in 3,329 subjects studied up until 2003) [14]. 

The use of super controls has been criticized by some on the grounds that their relatives will have rates of co-morbid disorders lower than that in the general population and may produce spurious co-aggregation of disorders within families. This argument is valid only if the same psychopathology that is removed from the control group is *not* excluded from among the probands and their relatives. 

This provides the rationale to encourage others to begin to carefully select true controls especially when dealing with complex traits such as RDS involving a number of associated gene polymorphisms [88,147,148,149]. 

Very few behaviors depend upon a single gene. Complexes of genes (polygenic) drive most of our heredity-based actions [150,151,152,153,154], suggesting that genetic panels or algorithms organized into genetic indexes, such as GARS may be valuable clinically to determine risk. Certainly abnormal functions of these brain systems can be due to specific genetic factors interacting with environmental factors [118,119,120,122,149,150,152,153,154]. Understanding the interactions of these components is likely to lead to better treatment.

Because our study began in 1999 when less was known about dopaminergic genes, we did not genotype subjects for the following: D3–5 receptor genes, the MAO-A or MAO-B, or catechol *O*-methyltransferase. However, those will be analyzed in subsequent experiments. When we started these experiments, the specific roles of the dopaminergic genes in brain function remained inconclusive due to the lack of completely selective ligands that could distinguish between the members of the D1-like and D2-like DA receptor families. However, today we are making rapid progress distinguishing among the various DA receptors (see [96]). Our findings, while suggestive, must be interpreted with caution. In terms of inclusion/exclusion criteria, since this was a generational study, all subjects were included in the study without bias.

### 4.3. Interactive Environmental and Genetic Roles in RDS Behaviors

It is important to consider one of the most important new areas in neurobiology and genetics termed “epigenetics” and its role in RDS. Most recently, our group reviewed the epigenetics of ADHD and detailed the important interaction of environmental elements and gene expression [154]. We are cognizant of the simple mathematical equation P = G + E. whereby, any phenotype (RDS included) is equal to both one’s genome and environmental impact. The role of the contribution of genetics to illicit drug abuse was evaluated by van den Bree *et al*. [155] using structural equation, modeling genetic and environmental estimates and DSM-III abuse/dependence for sedatives, opioids, cocaine, stimulants, and cannabis, as well as any other illicit drugs. The authors found genetic influences for most measures, which were strongest for males and for clinical diagnoses of abuse/dependence compared to actual substance use. Most interestingly, common environmental influences played a greater role in use than abuse/dependence, suggesting that the severity of any RDS behavior may have a stronger genetic contribution relative to less severe forms. Moreover, this same group [156] in another study, among Caucasians with alcohol dependence characterized subtypes by differential loading on three dimensions: genetic, general environmental, and dyssocial environmental symptom scales. The mild subtype (60% of men and 66% of women) was distinguished by low mean scores on all three scales; the dyssocial subtype (24% of men and 20% of women) by low mean genetic and general environmental scores but high mean dyssocial environmental scores; and the severe subtype (16% of men and 14% of women) by high scores on the genetic and general environmental scales. Importantly, the severe subtype showed greater comorbid drug dependence and major depression, more treatment seeking, and a higher prevalence of parental alcoholism. Only the severe subtype showed a pattern of scale scores and clinical characteristics suggestive of substantial genetic influence. 

It is well known that environmental cues may induce relapse in drug dependent individuals. This phenomenon has been evaluated by Gerasimov *et al*. [157] to better understand the neurochemical mechanisms potentially mediating these cues by measuring NAc DA levels in animals exposed to environmental cues previously paired with cocaine administration. They found that in animals exposed to a cocaine-paired environment, NAc DA increased by 25%. In one study the intake of morphine altered the neurotransmitter turnover of DA differentially as function of passive compared non-passive infusion. Environmental contingent infusion compared to passive infusion of intravenous morphine significantly affected more brain regions up to 5 fold [158] suggesting impact of the environment on neurochemistry. The role of dopaminergic genes as a predictor of risk concerning personality traits has been positively identified in molecular genetic studies. Earlier work in our laboratory identified the relationship between schizoid avoidance [143] as well as impulsive and compulsive behaviors [159]. The work of The *et al*. [160] showing significantly higher frequency for the DRD2 TaqIA polymorphism among addicts (69.9%) compared to control subjects (42.6%; Fisher’s exact χ^2^, p < 0.05) is in agreement with our earlier findings. They also observed that the studied addicts had higher scores for novelty seeking and harm avoidance personality traits but lower scores for reward dependence when compared to control subjects. This has been further supported by the work of Kazantseva *et al*. [161] on personality traits in a sample of 652 healthy individuals (222 men and 430 women) of Caucasian origin (233 Russians and 419 Tatars) from Russia. The subjects’ personality traits were assessed with Eysenck Personality Inventory and the Temperament and Character Inventory-125). There were significant effects of ANKK1/DRD2 Taq1A on Neuroticism (p = 0.016) and of SLC6A3 rs27072 on Persistence (p = 0.021) in both genders. The association between ANKK1/DRD2 Taq1A A2/A2-genotype and higher Novelty Seeking and lower Reward Dependence was shown in men only.

Genetic and environmental influences also are important for the development of alcohol and drug dependence. Exposure to early life stress, has been shown to predict a wide range of psychopathology, including addiction. Enoch [162] has suggested that early life stress can result in permanent neurohormonal and hypothalamic-pituitary-adrenal axis changes, morphological changes in the brain, and gene expression changes in the mesolimbic DA reward pathway, all of which are implicated in the development of addiction. However, he further emphasized that a large proportion of children who have experienced even severe early life stress do not develop psychopathology, indicating that mediating factors such as gene-environment interactions and family and peer relationships are important for resilience. Most interestingly, Israel researchers Raz and Berger [163] published convincing evidence for the role of social interaction as it related to intake of morphine in animals. Specifically, adult Wistar rats housed in short-term isolation (21 days) consumed significantly more morphine solution (0.5 mg/mL) than rats living in pairs, both in one-bottle and in two-bottle tests. They also found that as little as 60-min of daily social-physical interaction with another rat was sufficient to completely abolish the increase in morphine consumption in socially restricted animals. Accordingly, these results indicate that environmental and situational factors influence drug intake in laboratory rats as they do in humans.

Over many years of study, the consensus of the literature has suggested that prevention of drug-seeking relapse could be attenuated by enriched environments. For example, a recent French study by Chauvet *et al*. [164] showed, in animals, the potential “curative” influence of enriched environments in reducing cocaine-induced craving effects, thereby highlighting the importance of positive life conditions in facilitating abstinence and preventing relapse to cocaine addiction.

There is no doubt that enriched life experiences, as well as reduced early prenatal and post natal stress, have impacts on impulsivity. In addition, reduced release of mesolimbic DA affected both by genes and by the environment, play a significant role in craving behavior and more importantly in relapse [165,166,167,168,169] as well as many RDS behaviors [170,171,172,173,174,175,176,177,178,179,180,181,182,183,184,185,186,187,188,189,190,191,192,193] 

### 4.4. From Bench to Bedside: Clinical Utility of RDS

Historically, addictive disorders were categorized by the drug the patient was abusing, rather than the underlying neurocontrol circuitry that was being affected [194]. Alcohol and drug dependent individuals were considered to differ so substantially that treatment programs, as well as funding sources and regulatory agencies were distinct and separate. If insurance coverage was available, it was often restricted to the treatment of alcoholism, so that drug addicts often lied about their drug of choice in order to get admitted. Some methadone maintenance programs allowed drug addicts to drink, despite the fact that the leading cause of death at that time prior to viral infections was alcoholic cirrhosis. Self-help groups were organized around the drug of choice, such that if a person with a drug problem went to an Alcoholics Anonymous (AA) meeting, they were told that the meeting was for alcoholic-dependent individuals only and were asked to leave. Many polysubstance abusers who attended AA because they could not relate to Narcotics Anonymous (NA), learned not to volunteer their drug history. Patients on buprenorphine or methadone are still refrained from sponsoring or speaking at NA meetings, secondary to the belief that they are not abstinent (http://www.na.org/?ID=bulletins-bull29). 

Over the last 30 years the situation has improved because of our understanding of multiple addictive disorders as a brain disease [195,196,197,198,199,200,201,202,203,204,205,206]. Many programs have some treatment content on the medical aspects of addiction, although often it remains as an add-on without explicit integration into the other treatment suggestions. Often these presentations are provided by non-physicians in a treatment system that lacks a physician component in their multidisciplinary team, or depends upon medical referral to another agency or provider.

Only a handful of treatment centers have utilized the neurobiology of addiction as an integral part of patient education with explicit integration of RDS into all treatment content especially as it relates ADHD, Posttraumatic stress disorder and drug dependence [207,208,209,210,211,212,213]. This section of this paper will share how RDS can assist in patient education and acceptance of treatment recommendations including participating in self help and use of medication assisted therapy. These recommendations are organized around the American Society of Addiction Medicine’s (ASAM) six dimensions of care [214] and by treatment chronology.

ASAM’s six dimensions help to classify and assess treatment need and placement. They are: intoxication and withdrawal potential; biomedical conditions and complications; emotional behavioral conditions and complications; readiness for change; relapse potential; and recovery environment. Dimension 1 includes the substance use history, the extent of tolerance and physical dependence and the extent of polysubstance use. Clinicians are concerned about the extent of neuroadaptive changes and the severity of the addiction, but patients usually focus upon their drug of choice as the primary focus of intervention. We frequency hear from patients, “I came here to stop using cocaine, not alcohol and marijuana”. By reviewing the anatomy of control circuitry, the common pathway of DA release, and the concept of RDS as an inherited and acquired change in sensitivity and calibration of control circuitry, patients can now move from viewing the treatment providers as prohibitionists to seeing their concerns as medically based. Showing how DA increases in the NAc after natural rewards and by use of alcohol and drugs, patients understanding of the risk of cross addiction improves. Warning patients about continued use of substances that affect reward circuitry is coupled with drug testing to measure patient treatment adherence, especially when the program is providing detoxification and stabilization during early recovery. Documenting by patient history and drug testing that many patients have simultaneous poly substance use and dependence will allow for the program to document the medical necessity for testing and the choice of expanded panels to capture patients attempting to continue drug use that is not detectable by traditional testing. When testing for synthetic cannabinoids became available, we found that over 50% of patients in our adolescent IOP were getting high despite negative THC levels. 

Because of the high percentage of patients with co-occurring disorders (ASAM Dimension 2, medical, and Dimension 3, emotional/behavioral conditions and complications), use of RDS as a primary treatment concept allows patients to understand their biological differences and the role of inheritance. Over 80% of patients in our outpatient program have a positive family history of alcoholism and drug addiction, and often as children promised themselves that they would not grow up to be like their parents. Other patients suffer from binge eating, compulsive gambling, “shopaholism”, *etc*. A strong emphasis on the genetic aspects of RDS assists the patient in reducing guilt and shame and understanding how they could become something that they never planned on. This strengthens the importance of not over-relying on willpower and learning as primary treatment methods, and provides a medical justification of the counselors and sponsors recommendations. Acknowledging patients’ anhedonia during early recovery and incorporating thoughts, feelings and behaviors that increases reward, provides a therapeutic alliance and a potential acceptance by patients of treatment recommendations for proper nutrition, exercise, medications and other enjoyable activities.

By explaining the association with other disorders, RDS allows for the program and the patient to utilize standardized screening techniques for conditions such as ADD, gambling, aberrant sexual behavior as well as for personality profiles of aggressive and antisocial behaviors. Diagnosing patients with an associated condition(s) allows for education and early treatment. We have frequently observed impulsive behavior result in relapses during early recovery, as well as interfering with the quality of life and improved workplace functioning. Treating ADD earlier in recovery can be challenging, especially if the patient is focused upon becoming drug free and views any medications for ADD as part of the addictive process. Explaining how early treatment can help restore neurochemical balances provides a medical explanation that differentiates getting high from experiencing improved reward responsiveness to natural stimuli.

Not only is RDS helpful for the problems associated with ASAM Dimensions 1 through 3, but it also is most important in educating the patient about their disease (ASAM Dimension 4) and directly confronting altering cognition and defense mechanisms such as denial, rationalization, and justification. By explaining the pathophysiology of RDS, patients’ understanding of their illness improves significantly, especially in appreciating the subtle details of therapists’ recommendations. It is important for clinicians to explicitly connect RDS concepts with AA sayings and slogans so that the patient can understand why and how self-help works and is not simply an “option” in treatment. 

Relapse prevention (ASAM Dimension 5) can now be viewed as the processes of altered sensitivity and calibration of neuroadaptive circuitry, which affects hedonic tone, cue reactivity, and executive functioning. Warning patients that entering high risk situations during early recovery (the patient who frequents bars and who decides that it’s OK to play pool with his friends at the bar and drink soda), is not simply a matter of choice, but represents unintentional pressure upon altered circuitry. Understanding RDS concepts and the rate of restoration of recovery based neuroadaption allows for the patient to understand that the therapist recommendation is not punitive but protective. Many addicts do not understand that once the alcohol and drugs leave the body that the brain takes a long time to recover and may only reset itself to a genetically determined pre-morbid state. RDS emphasis the dynamics of neuroadaption of recovery and provides hope that improvement is possible, but at a rate of change that is dependent upon the amount of targeted treatment effort and the rate limiting steps from a biological perspective.

ASAM Dimension 6 (recovery environment) emphasizes the environmental impact on treatment effectiveness and encompasses the interactivity of gene expression by environment factors. Patients tend to underestimate the power of the environment in influencing outcome and tend to justify maintenance of their old relationships and living situations as an independent factor. Once patients understand the bidirectional aspect of the recovery environment, their motivation for making difficult changes can be modified by these biologically based factors. 

## 5. Limitations, Caveats and Future Directions

While we agree with the work of Sussman *et al*. [215] and his eloquent PACE model in terms of drug specificity based on many environmental elements, we further suggest that neurochemistry and certain specific genetic polymorphisms may lead to a non-specific hierarchical list of drugs of abuse and behaviors having a common neuro-chemical mechanism such as dopamine release in the NAc. However, this so called RDS phenotype is significantly impacted by availability of drugs,; awareness of drug availability, specific preference for one type over another type (depressant *vs*. stimulant), among other factors. 

Additional studies are required prior to any definitive interpretation of these data. We encourage other investigators to extend this work by analyzing many more families for hypodopaminergic genotypes. This should include D1–D5 receptors, MOA-A,B, COMT, as well as other reward genes. Certainly studies involving larger populations even in a few families over many generations (if possible) would strengthen this potentially important concept. Our laboratory is continuing our pursuit to enhance our knowledge base by carrying out linkage analysis to couple hypodopaminergic gene function with RDS behaviors. We encourage other investigators to perform similar experiments. 

## 6. Conclusions

While there have been other reports that help establish the RDS concept [169,170,171], additional research from multiple laboratories is warranted. The results summarized herein provide preliminary support for the hypothesis that dopaminergic genes, in particular the DRD2 and DAT1 polymorphisms, are significantly associated with the reward-dependent traits [172]. Although based on limited sample sizes, our findings may have direct implications for both the diagnosis and targeted treatment of RDS behaviors by analyzing the association of these dopaminergic genes and RDS behaviors in the form of proprietary algorithms or GARS. This research underscores the potential involvement of at least D2 receptor dysfunction as an important genetic antecedent to addiction as a disease. In keeping with the notion of common neurogenetic mechanisms, for impulsive, compulsive and addictive disorders, we propose that RDS is a basic phenotype covering many reward behaviors and pertinent psychiatric disorders (including spectrum disorders and Posttraumatic Stress Disorder) that should be included in the future in DSM as a genetic umbrella for many psychiatric diagnoses. 

While we are still evaluating these results using linkage analysis (requiring a different set of statistics), our present results suggest a paradigm shift in thinking about selecting appropriate phenotypes (controls and experimental) for reward dependence behaviors (due to reward deficiency). Further confirmation of these results should provide an impetus for appropriate and careful selection and screening of controls especially in reward dependence association studies in order to reduce the possibility of spurious outcomes (e.g., [173,211]).
